# Resource utilization and outcomes of inpatient eyelid reconstruction in facial palsy patients: A 20-year HCUP-NIS study

**DOI:** 10.1016/j.jpra.2026.09.003

**Published:** 2026-09-12

**Authors:** Cosima C. Hoch, Tobias Niederegger, Emre Karakas, Douglas Henderson, Thomas Schaschinger, Dilan Gucuk, Christian Festbaum, Jakob Fenske, Alexandre G. Lellouch, Leonard Knoedler, Andreas Kehrer

**Affiliations:** a Department of Otolaryngology, Head and Neck Surgery, TUM School of Medicine and Health, Technical University of Munich (TUM), Munich, Germany; b University of Heidelberg, Medical Faculty, Heidelberg, Germany; c Faculty of Medicine, Université Paris Cité, Paris, France; d Department of ENT and Head and Neck Surgery, Lariboisière Hospital, AP-HP, Paris, France; e Department of Visceral Surgery, Lausanne University Hospital (CHUV), Lausanne, Switzerland; f Division of Plastic and Reconstructive Surgery, University Hospital Regensburg, Germany; g Charité–Universitätsmedizin Berlin, corporate member of Freie Universität Berlin and Humboldt-Universität zu Berlin, Department of Oral and Maxillofacial Surgery, Mittelallee 2, Berlin, 13353, Germany; h Division of Plastic and Reconstructive Surgery, Cedars-Sinai Medical Center, Los Angeles, CA, USA; i Vascularized Composite Allotransplantation Laboratory, Massachusetts General Hospital, Harvard Medical School, Boston, MA, USA; j Innovative Therapies in Haemostasis, INSERM UMR-S 1140, University of Paris, Paris, 75006, France

**Keywords:** Facial Palsy, Eyelid Reconstruction, Inpatient Care, Resource Utilization, HCUP-NIS

## Abstract

**Background:**

Facial palsy–related lagophthalmos can threaten ocular surface integrity and may require surgical eyelid management.

**Methods:**

We conducted a retrospective cross-sectional analysis of adult inpatients in the Healthcare Cost and Utilization Project National/Nationwide Inpatient Sample from 2002 to 2021 with facial nerve disorder diagnosis codes and concurrent ICD-coded eyelid procedures. Demographics, hospital characteristics, length of stay, discharge disposition, charges, complications, comorbidity burden, and age- and income-stratified differences were analyzed.

**Results:**

We identified 1,839 inpatients with a median age of 60 years; 48.0% were female. Median length of stay was 4 days, median charges were $48,696, and patients underwent a mean of 4.96 coded procedures per admission. Complications were coded in 26.7% of cases. Older patients had longer hospitalizations, greater comorbidity burden, and higher complication rates, but lower median charges. Among patients with available income data, low- and high-income groups had similar length of stay and charges, despite differences in race, payer status, and geographic distribution.

**Conclusions:**

Inpatients with facial nerve disorders undergoing ICD-coded eyelid reconstruction represented a medically complex cohort with substantial hospital utilization and frequent coded complications.

## Background

Facial nerve palsy is a complex condition that compromises both the functional and aesthetic integrity of the face.^1^^,^^2^ It affects an estimated 15 to 40 individuals per 100,000 each year and may arise from a broad spectrum of etiologies, including Bell’s palsy, traumatic injury, neoplastic disease, and iatrogenic injury, congenital conditions, and infectious causes.^3^^,^^4^ Although many patients recover partially or completely, a substantial proportion experience persistent deficits. One of the most serious sequelae is ocular morbidity, particularly lagophthalmos, the inability to fully close the eyelids. This dysfunction can lead to exposure keratopathy, corneal desiccation, and ultimately irreversible visual loss. Without timely intervention, patients are at risk for chronic ocular discomfort, recurrent infection, and vision impairment, all of which substantially diminish quality of life.^5^, ^6^, ^7^ Beyond functional limitations, facial asymmetry and disfigurement carry profound psychosocial consequences, affecting self-image, emotional well-being, and social interactions.^8^, ^9^, ^10^

A spectrum of options exists to manage facial palsy–related lagophthalmos, ranging from conservative measures such as lubricants and temporary tarsorrhaphy to definitive surgical approaches.^11^, ^12^, ^13^ Surgical eyelid procedures represent the mainstay of long-term ocular surface protection and include passive upper eyelid loading, lower eyelid tightening procedures such as lateral tarsal strip or canthoplasty, and more complex static or dynamic reanimation techniques involving canthal tendon manipulation, fascial slings, or neurotized flaps.^14^, ^15^, ^16^

Despite the central role of these operations in restoring basic protective eyelid function, there is a notable lack of large-scale data on their real-world utilization. Most published studies comprise small case series or single-center cohorts, limiting external validity. A broader understanding of which patients undergo these procedures, in what clinical settings, and at what cost is critical for optimizing care pathways and promoting equitable access.^17^^,^^18^

To address this gap within the limitations of inpatient administrative data, we performed a nationwide analysis of facial palsy patients undergoing ICD-coded inpatient eyelid procedures using the Healthcare Cost and Utilization Project Nationwide Inpatient Sample (HCUP-NIS). Because the ICD-10-PCS strategy available for this analysis was restricted to eyelid replacement code families, this study should be interpreted as characterizing a narrowly defined inpatient eyelid replacement/reconstruction cohort rather than the full spectrum of facial palsy–related eyelid surgery. We sought to describe demographic and clinical profiles, hospital-level patterns, length of stay, discharge disposition, complications, and hospital charges in this high-acuity inpatient subgroup.

## Methods

### Data source

We extracted data from the HCUP-NIS, which captures a representative 20% sample of all hospital discharges in the United States, encompassing more than 7 million discharges annually.^19^^,^^20^ The dataset includes detailed information on patient demographics, diagnoses and procedures (ICD-9-CM and ICD-10-CM/PCS), hospital characteristics, outcomes, and cost metrics. For this study, we used discharges between 2002 and 2021, which spans the pre- and post-transition periods from ICD-9 to ICD-10 coding systems.

### Patient selection and procedure classification

Inclusion criteria comprised adult patients aged ≥18 years with a documented diagnosis of facial nerve disorder, defined by ICD-9-CM code 351.0 or ICD-10-CM code family G51, in conjunction with a corresponding procedural code for eyelid surgery. Eyelid procedures were identified using the ICD-9-CM procedure code prefix 08, corresponding to operations on the eyelids. For the ICD-10-PCS era, the available extraction strategy was limited to the eyelid replacement code families 08RN, 08RP, 08RQ, and 08RR, corresponding to replacement procedures involving the upper or lower eyelid. Patients without identifiable procedure codes or those not meeting the specified diagnostic and procedural criteria were excluded from the analysis. Importantly, ICD-10-PCS procedure coding distinguishes replacement from other root operations, including insertion, repair, reposition, and supplement. Therefore, common facial palsy–related eyelid procedures such as upper eyelid loading with gold or platinum weights, tarsorrhaphy, lateral tarsal strip, canthoplasty, and other lid-tightening procedures may not have been captured by the ICD-10-PCS codes used in this analysis. The resulting cohort should therefore be interpreted as a narrowly defined inpatient eyelid replacement/reconstruction cohort rather than a comprehensive sample of all surgical eyelid management in facial palsy.

### Variables collected

We collected data on patient demographics, including age, sex, race or ethnicity, and ZIP-code-based income quartile; hospitalization characteristics, such as length of stay (LOS), discharge disposition, and total charges; and comorbidity burden, assessed using the number of chronic conditions and diagnostic codes. Procedure details were obtained from up to 25 procedural fields per admission, and hospital-level factors, including region and teaching status (when available), were also recorded.

### Statistical analysis

Continuous variables were summarized as mean ± SD and median with IQR and compared using Welch’s t-tests. Categorical variables were summarized as frequencies and percentages and compared using Pearson’s chi-square tests. Income-stratified analyses were restricted to patients with available ZIP-code income quartile data; Q1–Q2 were categorized as low income and Q3–Q4 as high income. A p-value < 0.05 was considered statistically significant. Analyses were performed in R version 4.5.1.

## Results

### Patient demographics

Because ICD-10-PCS case identification was limited to eyelid replacement code families, the final cohort represents inpatients with facial nerve disorders undergoing ICD-coded eyelid replacement/reconstructive procedures, not all patients undergoing facial palsy–related eyelid surgery. The final cohort comprised 1,839 patients with a mean age of 57.3 ± 19.7 years (median 60, IQR 46–72), and n = 883 (48.0%) were female. Most patients were White, and Medicare and private insurance were the predominant payers. Income quartiles were reported for just over half the cohort, with n = 259 (14.1%) in the highest quartile and n = 231 (12.6%) in the lowest, while n = 869 (47.3%) lacked income data. Insurance coverage was most commonly Medicare (n = 762; 41.4%) or private insurance (n = 707; 38.4%). Medicaid covered n = 167 (9.1%), whereas n = 81 (4.4%) were self-paying. Geographic information was unavailable for most individuals (n = 1,602; 87.1%); among those with known residence, metropolitan areas with ≥1 million inhabitants accounted for n = 62 (3.3%) in central counties and n = 70 (3.8%) in fringe counties (Table 1).

**Table 1:** Patient demographics and hospitalization data.

**Variable**		**Total cohort (n = 1,839)**
**Epidemiological data**		
Age [years ± SD]		57.26 ± 19.69
Age [median + (IQR)]		60 (46 - 72)
Female		883 (48.0%)
Ethnicity		
	White	1,144 (62.2%)
	Black	122 (6.6%)
	Hispanic	158 (8.6%)
	Asian or pacific	45 (2.4%)
	Native American	10 (0.5%)
	Other	70 (3.8%)
	Unknown	259 (14.1%)
**Income quartile**		
	Low (Q1)	231 (12.6%)
	Mid-low (Q2)	237 (12.9%)
	Mid-high (Q3)	243 (13.2%)
	High (Q4)	259 (14.1%)
	Unknown	869 (47.3%)
**Primary payer/insurance**		
	Medicare	762 (41.4%)
	Medicaid	167 (9.1%)
	Private insurance	707 (38.4%)
	Self-pay	81 (4.4%)
	No charge	18 (1.0%)
	Other	100 (5.4%)
	Unknown	4 (0.2%)
**Patient location**		
	Central counties in metro areas of ≥1 million population	62 (3.3%)
	Fringe counties in metro areas of ≥1 million population	70 (3.8%)
	Counties in metro areas of 250,000-999,999 population	48 (2.6%)
	Counties in metro areas of 50,000-249,999 population	20 (1.1%)
	Micropolitan counties	19 (1.0%)
	Not metropolitan or micropolitan counties	18 (1.0%)
	Unknown	1602 (87.1%)
**Hospitalization data**		
Length of stay [days ± SD]		7.55 ± 10.95
Length of stay [median (IQR)]		4 (2 - 9)
Number of total ICD-10 procedures [mean ± SD]		4.96 ± 3.02
Charges [mean US-Dollar ± SD]		$71,971.55 ± $81,218.06
Charges [median (IQR)]		$48,696 ($25,838.25 - $86,676)
**Manner of discharge**		
	Routine	584 (31.8%)
	Transfer to Short-term Hospital	12 (0.7%)
	Transfer Other [including SNF, ICF]	218 (11.9%)
	Home Health Care	183 (10.0%)
	Against Medical Advice	2 (0.1%)
	Died	3 (0.2%)
	Unknown	837 (45.5%)
**Hospital location**		
	New England	11 (0.6%)
	Middle Atlantic	66 (3.6%)
	East North Central	52 (2.8%)
	West North Central	37 (2.0%)
	South Atlantic	69 (3.8%)
	East South Central	17 (0.9%)
	West South Central	42 (2.3%)
	Mountain	21 (1.1%)
	Pacific	53 (2.9%)
**Complications & comorbidities**		
	Complications	491 (26.7%)
	Number of Diagnoses [mean ± SD]	9.19 ± 4.68
	Number Chronic Conditions [mean ± SD]	4.32 ± 2.84

### Hospitalization parameters

Hospital resource utilization was substantial. Patients experienced a median hospital stay of 4 days (IQR 2–9; mean 7.55 ± 10.95 days). Median hospital charges were $48,696 (IQR $25,838–$86,676; mean $71,971.55 ± $81,218.06). The difference between mean and median charges reflects the right-skewed distribution of hospital charges, in which a smaller number of high-charge admissions increases the mean. On average, individuals underwent 4.96 ± 3.02 ICD-10–coded procedures during their admission. Most patients were discharged in routine fashion (n = 584; 31.8%), while others required transfer to another facility (n = 218; 11.9%) or home health services (n = 183; 10.0%). A small number left against medical advice (n = 2; 0.1%) or died during hospitalization (n = 3; 0.2%). Discharge disposition was undocumented for a large proportion of cases (n = 837; 45.5%). Procedures were distributed across US Census hospital divisions, with the South Atlantic (n = 69; 3.8%) and Middle Atlantic (n = 66; 3.6%) divisions accounting for the highest proportions among recorded divisions. Complications occurred in 26.7% (n = 491) of the cohort, who presented with an average of 9.19 ± 4.68 diagnoses and 4.32 ± 2.84 chronic conditions (Table 1).

### Subgroup comparison of younger and older patients

Patients were stratified into younger and older groups using the cohort’s median age of 60 years as the cutoff. Significant demographic and clinical differences were observed between younger (n = 892; 48.5%) and older patients (n = 947; 51.5%). Age groups differed markedly in racial distribution (p < 0.001), with older patients more frequently identifying as White (69.8% vs. 54.1%) and younger patients showing higher proportions of Hispanic (12.9% vs. 4.5%) and Black individuals (8.2% vs. 5.2%). Primary payer patterns also varied substantially (p < 0.001): Medicare predominated among older adults (72.1%), whereas private insurance (58.3%) and Medicaid (15.4%) were more common in younger patients. Older patients experienced longer hospitalizations (6.65 ± 7.45 vs. 3.93 ± 7.90 days, p < 0.001) yet incurred lower mean charges ($64,449 vs. $80,018, p < 0.001). Discharge disposition also differed significantly (p < 0.001), with younger patients more frequently discharged home routinely (35.5% vs. 28.2%) and older individuals more often transferred to skilled nursing or intermediate care (16.4% vs. 7.1%). Older adults further exhibited higher complication rates (28.8% vs. 24.4%, p = 0.03) and a greater comorbidity burden, reflected in significantly more diagnoses (10.13 ± 4.57 vs. 8.18 ± 4.55, p < 0.001) and chronic conditions (4.95 ± 2.80 vs. 3.61 ± 2.70, p < 0.001) (Table 2).

**Table 2:** Subgroup analysis of young and old patients stratified through the median age of the patient cohort.

**Variable**		**Young (<60 years)**	**Old (≥60 years)**	***p - value***
**n**		892 (48.5%)	947 (51.5%)	
**Patient Socioeconomic & Demographic Data**				
Age [years ± SD]		41.16 ± 14.84	72.44 ± 8.39	
Age [median + (IQR)]		45 (32 - 53)	71 (65 – 79)	
Female		446 (50%)	437 (46.1%)	0.09
**Ethnicity**				**< 0.001**
	White	483 (54.1%)	661 (69.8%)	
	Black	73 (8.2%)	49 (5.2%)	
	Hispanic	115 (12.9%)	43 (4.5%)	
	Asian or Pacific Islander	28 (3.1%)	17 (1.8%)	
	Native American	5 (0.6%)	5 (0.5%)	
	Other	46 (5.2%)	24 (2.5%)	
	Unknown	144 (16.1%)	148 (15.6%)	
**ZIP Code Income Quartile**				0.72
	Low (Q1)	103 (11.5%)	128 (13.5%)	
	Mid-Low (Q2)	116 (13.0%)	121 (12.8%)	
	Mid-High (Q3)	114 (12.8%)	129 (13.6%)	
	High (Q4)	115 (12.9%)	144 (15.2%)	
	Unknown	444 (49.8%)	425 (44.9%)	
**Expected Primary Payer**				**< 0.001**
	Medicare	79 (8.9%)	683 (72.1%)	
	Medicaid	137 (15.4%)	30 (3.2%)	
	Private Insurance	520 (58.3%)	187 (19.7%)	
	Self-pay	63 (7.1%)	18 (1.9%)	
	No Charge	16 (1.8%)	2 (0.2%)	
	Other	74 (8.3%)	26 (2.7%)	
	Unknown	3 (0.3%)	1 (0.1%)	
**Patient Location**				0.39
	Central counties in metro areas of ≥1 million population	28 (3.2%)	34 (3.6%)	
	Fringe counties in metro areas of ≥1 million population	26 (2.9%)	44 (4.6%)	
	Counties in metro areas of 250,000-999,999 population	12 (1.3%)	36 (3.8%)	
	Counties in metro areas of 50,000-249,999 population	7 (0.8%)	13 (1.4%)	
	Micropolitan counties	7 (0.8%)	12 (1.3%)	
	Not metropolitan or micropolitan counties	8 (0.9%)	10 (1.1%)	
	Unknown	804 (90.0%)	798 (84.3%)	
**Hospital Admission & Course Data**				
Length of Stay [days ± SD]		3.93 ± 7.90	6.65 ± 7.45	**< 0.001**
Length of Stay [median (IQR)]		4 (2 - 9)	4 (2 - 8)	
Number of Procedures [mean ± SD]		5 ± 2.96	4.95 ± 3.17	0.73
Charges [mean US-Dollar ± SD]		$80,017.78 ± $96,817.36	$64,448.71 ± $ 62,465.43	**< 0.001**
Charges [median (IQR)]		$50,808 ($27,513.5 - $92,321.75)	$ 46,932.5 ($ 23,749.5 - $ 81,693)	
**Manner of Discharge**				**< 0.001**
	Routine	317 (35.5%)	267 (28.2%)	
	Transfer to Short-term Hospital	8 (0.9%)	4 (0.4%)	
	Transfer Other [including SNF, ICF]	63 (7.1%)	155 (16.4%)	
	Home Health Care	72 (8.1%)	111 (11.7%)	
	Against Medical Advice	2 (0.2%)	0 (0.0%)	
	Died	0 (0.0%)	3 (0.3%)	
	Unknown	430 (48.2%)	407 (43.0%)	
**Hospital Division**				0.66
	New England	3 (0.3%)	8 (0.8%)	
	Middle Atlantic	29 (3.3%)	37 (3.9%)	
	East North Central	17 (1.9%)	35 (3.7%)	
	West North Central	17 (1.9%)	20 (2.1%)	
	South Atlantic	30 (3.4%)	39 (4.1%)	
	East South Central	10 (1.1%)	7 (0.7%)	
	West South Central	20 (2.2%)	22 (2.3%)	
	Mountain	8 (0.9%)	13 (1.3%)	
	Pacific	22 (2.5%)	31 (3.3%)	
**Complications & Comorbidities**				
	Complications	218 (24.4%)	273 (28.8%)	**0.03**
	Number of Diagnoses [mean ± SD]	8.18 ± 4.55	10.13 ± 4.57	**< 0.001**
	Number Chronic Conditions [mean ± SD]	3.61 ± 2.70	4.95 ± 2.80	**< 0.001**

### Comparison of low- and high-income patients

Income data were available for 970 patients, including 468 low-income and 502 high-income patients. Age and sex were comparable between groups. Racial and ethnic distribution differed significantly (p = 0.001), with high-income patients more frequently identifying as White and low-income patients showing higher proportions of Black and Hispanic individuals. Payer status also differed (p < 0.001), with Medicaid more common in the low-income group and private insurance more common in the high-income group. High-income patients more frequently resided in large metropolitan areas, whereas low-income patients were more often represented in micropolitan and non-metropolitan counties. Hospitalization parameters were largely comparable: median length of stay was 4 days in both groups, median charges were similar ($62,702 vs. $62,983), procedural counts and discharge patterns did not differ significantly, and complications were coded in 28.0% versus 30.1% of patients (p = 0.47). Low-income patients had a higher mean number of diagnoses, while chronic conditions did not differ significantly (Table 3).

**Table 3:** Subgroup analysis of patients with available ZIP-code income data, stratified into low- and high-income groups.

**Variable**		**Low Income**	**High Income**	***p - value***
**n**		468 (48.2%)	502 (51.8%)	
**Patient Socioeconomic & Demographic Data**				
Age [years ± SD]		58.06 ± 19.06	58.38 ± 18.91	0.79
Age [median (IQR)]		61 (49 - 71)	61 (47 - 73)	
Female		217 (46.6%)	252 (50.2%)	0.23
**Ethnicity**				**0.001**
	White	283 (60.5%)	367 (73.1%)	
	Black	46 (9.8%)	27 (5.4%)	
	Hispanic	44 (9.4%)	38 (7.6%)	
	Asian or Pacific Islander	9 (1.9%)	15 (3.0%)	
	Native American	6 (1.3%)	0 (0.0%)	
	Other	15 (3.2%)	17 (3.4%)	
	Unknown	65 (13.9%)	38 (7.6%)	
**Expected Primary Payer**				**<0.001**
	Medicare	204 (43.6%)	219 (43.6%)	
	Medicaid	56 (12.0%)	23 (4.6%)	
	Private Insurance	150 (32.1%)	212 (42.2%)	
	Self-pay	19 (4.1%)	13 (2.6%)	
	No Charge	6 (1.3%)	3 (0.6%)	
	Other	31 (6.6%)	31 (6.2%)	
	Unknown	2 (0.4%)	1 (0.2%)	
**Patient Location**				**<0.001**
	Central counties in metro areas of ≥1 million population	26 (5.6%)	34 (6.8%)	
	Fringe counties in metro areas of ≥1 million population	12 (2.6%)	58 (11.6%)	
	Counties in metro areas of 250,000-999,999 population	16 (3.4%)	31 (6.2%)	
	Counties in metro areas of 50,000-249,999 population	12 (2.6%)	8 (1.6%)	
	Micropolitan counties	17 (3.6%)	2 (0.4%)	
	Not metropolitan or micropolitan counties	14 (3.0%)	3 (0.6%)	
	Unknown	371 (79.3%)	366 (72.9%)	
**Hospital Admission & Outcome Data**				
Length of Stay [days ± SD]		7.18 ± 10.1	6.62 ± 9.53	0.38
Length of Stay [median (IQR)]		4 (2-9)	4 (2-7)	
Number of Procedures [mean ± SD]		5.08 ± 3.29	5.41 ± 3.39	0.12
Charges [mean US-Dollar ± SD]		$86,535.13 ± $87,263.72	$87,251.12 ± $86,377.41	0.90
Charges [median (IQR)]		$62,702 ($35,665.5 - $104,683.5)	$62,983 ($34,243 - $108,900)	
**Manner of Discharge**				0.48
	Routine	263 (56.2%)	298 (59.4%)	
	Transfer to Short-term Hospital	4 (0.9%)	8 (1.6%)	
	Transfer Other [including SNF, ICF]	105 (22.4%)	105 (20.9%)	
	Home Health Care	92 (19.7%)	90 (17.9%)	
	Against Medical Advice	2 (0.4%)	0 (0.0%)	
	Died	2 (0.4%)	1 (0.2%)	
	Unknown	0 (0.0%)	0 (0.0%)	
**Hospital Division**				**<0.001**
	New England	2 (0.4%)	9 (1.8%)	
	Middle Atlantic	20 (4.3%)	44 (8.7%)	
	East North Central	27 (5.8%)	26 (5.2%)	
	West North Central	19 (4.1%)	17 (3.4%)	
	South Atlantic	35 (7.5%)	40 (8.0%)	
	East South Central	12 (2.6%)	3 (0.6%)	
	West South Central	26 (5.6%)	16 (3.2%)	
	Mountain	14 (3.0%)	7 (1.4%)	
	Pacific	14 (3.0%)	39 (7.8%)	
**Complications & Comorbidities**				
	Complications	131 (28.0%)	151 (30.1%)	0.47
	Number of Diagnoses [mean ± SD]	10.91 ± 5.1	10.18 ± 5.04	**0.02**
	Number Chronic Conditions [mean ± SD]	4.49 ± 2.88	4.2 ± 2.78	0.11

Income-stratified analyses excluded patients with missing ZIP-code income quartile data (n = 869). Low income includes Q1–Q2; high income includes Q3–Q4. The threshold between groups corresponds to the boundary between Q2 and Q3 of the HCUP-NIS ZIP-code income quartile variable; absolute dollar ranges vary by year.

## Discussion

Using two decades of HCUP-NIS data, we characterized a narrowly defined inpatient cohort of facial nerve disorder patients undergoing ICD-coded inpatient eyelid reconstruction and quantified associated hospital utilization and charges. In this cohort of 1,839 adult inpatients, hospital stays were prolonged, charges were substantial, coded complications were frequent, and comorbidity burden was high. Importantly, complication coding in administrative datasets reflects overall inpatient morbidity and cannot be attributed directly to the eyelid procedure itself. Given the administrative and observational nature of the dataset, this study is descriptive and does not allow causal inference. These findings suggest that the patients captured by this administrative coding strategy represent a medically complex inpatient subgroup, likely undergoing eyelid intervention in the context of broader reconstructive, traumatic, oncologic, or neurologic care, rather than the typical elective outpatient eyelid-loading or eyelid-tightening procedures commonly described in facial palsy surgical series. Importantly, because the ICD-10-PCS strategy was limited to eyelid replacement code families, this study should not be interpreted as estimating the overall utilization of facial palsy–related eyelid surgery.

Resource utilization was substantial, reflected by prolonged LOS and high hospital charges. However, these values should be interpreted in the context of the inpatient-only NIS and the right-skewed distribution of hospital charges. For context, an HCUP-NIS study of autologous free-flap breast reconstruction reported the same median LOS of 4 days.^21^ In facial and craniofacial reconstructive cohorts, NIS analyses of microtia repair reported subgroup LOS estimates of 1.39–2.33 days and procedure costs rising from $25,897 in 2008 to $48,985 in 2015, whereas total alloplastic temporomandibular joint replacement had a mean LOS of 2.6 days and mean hospital charges of $108,709.^22^^,^^23^ These benchmarks indicate that our median LOS was comparable to free-flap breast reconstruction but longer than the reported facial and craniofacial values, while our median charges were lower than those reported for temporomandibular joint replacement and similar in magnitude to the 2015 microtia cost estimate. Direct comparisons remain limited by differences in patient age, case mix, study period, summary statistics, and cost/charge methodology. In contrast, much of the oculoplastic literature describes eyelid loading with gold or platinum weights as durable and commonly performed in elective outpatient or ambulatory settings.^24^, ^25^, ^26^, ^27^, ^28^, ^29^, ^30^, ^31^ This discrepancy likely reflects database capture: inpatient eyelid procedures may occur in patients admitted for tumor surgery, trauma, complex neurologic disease, concurrent reconstruction, or urgent ocular-surface protection. Even in eyelid-loading literature, complications such as inflammatory reactions, implant prominence/extrusion, and late tissue thinning have been reported and may be more consequential in medically fragile inpatients.^32^^,^^33^

We also observed a high “complication” rate (26.7%) in the inpatient cohort. This is higher than what many single-center series would suggest for isolated procedures such as lateral tarsal strip and limited tarsorrhaphy performed for paralytic ectropion, which are often described as safe and effective with low rates of major adverse events in appropriately selected patients.^34^^,^^35^ Prior studies focused on lower-lid paralytic ectropion describe durable symptom and position improvement with lateral tarsal strip-based approaches, including series reporting low recurrence and limited morbidity during follow-up.^34^^,^^36^^,^^37^ A plausible explanation is that complications coded during these admissions may reflect global inpatient morbidity driven by patient complexity rather than procedure-attributable events, particularly given the high baseline comorbidity burden and the likelihood that eyelid surgery may be only one of several procedures performed during hospitalization.

Age-stratified analyses showed that older patients (≥60 years) experienced longer hospitalizations, higher comorbidity burden, and slightly higher complication rates, yet lower mean charges than younger patients. Longer LOS and higher complication rates in older adults are consistent with broader perioperative literature showing that frailty and multimorbidity are associated with prolonged hospitalization and worse inpatient outcomes, including increased postoperative morbidity and functional decline.^38^, ^39^, ^40^ A key nuance is that our financial metric is “total charges,” not true costs or reimbursements. In HCUP, charges represent billed amounts and can diverge substantially from underlying costs; converting charges to estimated costs typically requires applying hospital-specific cost-to-charge ratios. Therefore, the lower charges observed among older patients could reflect differences in case mix (e.g., fewer high-charge ancillary services), payer-related billing patterns, hospital characteristics, or care pathways, rather than lower resource consumption.

Socioeconomic stratification revealed meaningful disparities in demographic composition, payer mix, and geographic access proxies between low- and high-income groups: low-income patients were more frequently Black or Hispanic and more commonly insured by Medicaid, whereas high-income patients were more often White, privately insured, and treated in large metropolitan regions; yet LOS and charges were similar across income groups. Because nearly half of the overall cohort lacked ZIP-code income data, income-stratified findings should be interpreted as applying only to the subset with available income information and not necessarily to the full cohort. The divergence in payer distribution suggests that access to surgical eyelid management within inpatient settings may partially mirror broader inequities in insurance coverage and referral pathways. This aligns with evidence of geographic and socioeconomic disparities in access to oculofacial plastic surgeons and with broader ophthalmic literature documenting inequities in eye care access and outcomes.^41^^,^^42^ Insurance-related barriers may be especially relevant for specialty access: recent analyses have reported Medicaid access limitations for eye care providers and reimbursement-related factors that could contribute to differential access and timeliness of subspecialty care.^43^^,^^44^ One hypothesis is that privately insured, higher-income patients may have greater access to outpatient oculoplastic services, meaning those captured in an inpatient dataset could represent a selective subset referred to tertiary centers or treated in urban high-capacity systems, while lower-income patients may present later or through different entry points (e.g., emergency or safety-net pathways) that still culminate in inpatient care when ocular risk becomes urgent.

## Limitations

This study has several limitations inherent to the use of administrative databases. First, the HCUP-NIS captures only inpatient hospitalizations, excluding the majority of eyelid procedures performed in outpatient or ambulatory settings. Consequently, our findings likely represent a high-acuity subset rather than the broader facial palsy population. Second, and most importantly, the ICD-10-PCS procedure-code strategy used for this analysis was limited to eyelid replacement code families (08RN, 08RP, 08RQ, 08RR). These codes capture a narrow procedural subset and do not comprehensively identify the full range of facial palsy–related eyelid procedures. Common procedures such as eyelid loading, tarsorrhaphy, lateral tarsal strip, canthoplasty, and other lid-positioning procedures may therefore be undercaptured. Because we were unable to re-extract the cohort using an expanded ICD-10-PCS strategy, the present findings should be interpreted as describing a selected inpatient eyelid replacement/reconstruction cohort rather than the epidemiology of all surgical eyelid management in facial palsy. This selection bias likely underestimates the true procedural volume and limits generalizability, particularly for common outpatient or ambulatory procedures. Third, complications in the NIS reflect all coded inpatient adverse events and may not directly relate to the eyelid procedure itself. Accordingly, the reported complication rate should be interpreted as a marker of overall inpatient morbidity rather than procedure-specific surgical morbidity. Given the observational and administrative nature of the dataset, this study is descriptive and cannot establish causal relationships between patient characteristics, procedures, complications, length of stay, or charges. Fourth, hospital charges reported in the database do not represent actual costs or reimbursements and vary by institution and payer. Fifth, the transition from ICD-9 to ICD-10 in 2015 may have influenced coding frequencies and comparability across years. Sixth, the database structure and available coding granularity did not allow robust comparisons across clinically meaningful subgroups, such as upper versus lower eyelid procedures; implant-bearing versus non-implant techniques, including platinum versus gold eyelid weights; and canthoplasty versus canthopexy, because these distinctions are not consistently captured in HCUP-NIS input fields and because the relevant procedural detail is encoded differently between the ICD-9 and ICD-10 coding systems. As a result, stratified analyses are further constrained by small subgroup sample sizes after coding-based case identification. Additionally, ZIP-code income quartile data were unavailable for 869 patients (47.3% of the cohort). Therefore, income-stratified analyses were restricted to patients with available income data, which may explain differences between the overall cohort charge distribution and the charge distributions observed in the income subgroups. This missingness may introduce selection bias and limits the generalizability of income-based comparisons. Lastly, granular clinical data such as facial palsy etiology, timing of surgery relative to facial nerve injury, and specific eyelid techniques are unavailable, limiting the ability to perform detailed risk-adjusted outcome analyses.

## Conclusions

Inpatients with facial nerve disorders undergoing ICD-coded eyelid replacement/reconstructive procedures represent a medically complex subgroup with substantial resource utilization, high comorbidity burden, and frequent coded complications. Because the ICD-10-PCS strategy was limited to eyelid replacement code families, these findings should not be interpreted as capturing the full spectrum of facial palsy–related eyelid surgery. Future studies integrating inpatient, outpatient, and ambulatory datasets with expanded coding strategies are needed to define utilization, costs, and disparities more accurately.

## Human ethics and consent to participate

Not applicable. The institutional review board at Cedars-Sinai Medical Center (Los Angeles, CA, USA) deemed this study exempt from formal review.

## Consent for publication

Not applicable.

## Clinical trial number

Not applicable.

## Funding

None.

## Availability of data and materials

The HCUP NIS data are available from the AHRQ HCUP Central Distributor subject to the applicable purchase and Data Use Agreement requirements; the authors are not permitted to redistribute the data.

## Declaration of competing interest

The authors declare that they have no competing interests.
